# Effect of dairy fat on plasma phytanic acid in healthy volunteers - a randomized controlled study

**DOI:** 10.1186/1476-511X-10-95

**Published:** 2011-06-10

**Authors:** Louise B Werner, Lars I Hellgren, Marianne Raff, Søren K Jensen, Rikke A Petersen, Tue Drachmann, Tine Tholstrup

**Affiliations:** 1 Department of Human Nutrition, Faculty of Life, University of Copenhagen, Frederiksberg 1958, Denmark; 2 Center for Biological Sequence Analysis, DTU Systems Biology, Technical University of Denmark, 2800 Lyngby, Denmark; 3 Department of Animal Health and Bioscience, Faculty of Agricultural Sciences, Aarhus University, 8830 Tjele, Denmark

**Keywords:** Phytanic acid, cow-feeding regime, absorption, fatty acid, total cholesterol, LDL cholesterol, HDL cholesterol, C-reactive protein, insulin, glucose

## Abstract

**Background:**

Phytanic acid produced in ruminants from chlorophyll may have preventive effects on the metabolic syndrome, partly due to its reported RXR and PPAR- α agonist activity. Milk from cows fed increased levels of green plant material, contains increased phytanic acid concentrations, but it is unknown to what extent minor increases in phytanic acid content in dairy fat leads to higher circulating levels of phytanic acid in plasma of the consumers.

**Objective:**

To investigate if cow feeding regimes affects concentration of plasma phytanic acid and risk markers of the metabolic syndrome in human.

**Design:**

In a double-blind, randomized, 4 wk, parallel intervention study 14 healthy young subjects were given 45 g milk fat/d from test butter and cheese with 0.24 wt% phytanic acid or a control diet with 0.13 wt% phytanic acid. Difference in phytanic acid was obtained by feeding roughage with low or high content of chlorophyll.

**Results:**

There tended to be a difference in plasma phytanic acid (P = 0.0730) concentration after the dietary intervention. Plasma phytanic acid increased significantly within both groups with the highest increase in control group (24%) compared to phytanic acid group (15%). There were no significant effects of phytanic acid on risk markers for the metabolic syndrome.

**Conclusions:**

The results indicate that increased intake of dairy fat modify the plasma phytanic acid concentration, regardless of cows feeding regime and the minor difference in dietary phytanic acid. Whether the phytanic acid has potential to affects the risk markers of the metabolic syndrome in human still remain to be elucidated.

**Trial Registration:**

ClinicalTrials.gov: NCT01343576

## Background

Phytanic acid is a multi branched-chain fatty acid formed through the metabolism of phytol, which is released from the chlorophyll molecule in the rumen of ruminant animals [1]. Hence, dairy-products, ruminant meat and also some marine fats are the only major dietary sources of phytanic acid or its metabolic precursor, phytol [2]. Dietary intake of phytanic acid have been suggested to be involved in both health- and disease promoting processes, thus some researchers have suggested that it can prevent diabetes and metabolic diseases, while others have suggested that it promotes development of prostate cancer [3,4].

The potential health-promoting properties is based on the fact that animal and in vitro studies have shown that phytanic acid might have preventive effects on metabolic dysfunctions, since it in animal studies increase expression of genes involved in fatty acid oxidation, enhance glucose uptake and metabolism in hepatocyte and potentially reduce metabolic efficacy through increased differentiation of brown adipocyte differentiation and expression of uncoupling protein-1 [5-7]. These effects have partly been explained by the PPAR and RXR agonist activity of phytanic acid [6,8-10]. Hence, phytanic acid may be an interesting, though so far overlooked, bioactive FA in dairy products with potential positive effects on metabolic function. However, the suggested involvement of phytanic acid in development of prostate cancer also needs to be taken into consideration. To be able to evaluate the potential risks and advantages of a dietary intake of phytanic acid, we need more data on how intake of phytanic acid containing foodstuffs affects levels of phytanic acid, as well as data on effects on metabolic markers in humans. Although earlier observational studies have found a significant correlation between dairy fat intake and plasma levels of phytanic acid [3,11,12] this is to over knowledge, the first controlled human intervention study in which the relation between dairy fat intake and plasma phytanic acid are directly established.

It is well known that the FA composition of milk fat is markedly influenced by the diet, especially by the amount and FA profile of fat in the cattle fodder [13-16]. Since phytanic acid is formed from metabolites of chlorophyll degradation, the content in milk fat is strongly dependent on the amount of chlorophyll-containing material in the feed [17,18], and will also change over the year depending on the content of green plant material in the cows diet. A study has shown that the phytanic acid concentration in milk fat increased from 0.15 mass% to 0.45 mass%, in cows fed grass only compared to a group fed a mixture of rye grass and maize silage [18] and it was recently shown that the concentration of phytanic acid is higher in organic than conventional dairy products [19].

In this study the test fat was made from milk from one herd divided into two groups that were fed either "green" silage aiming after a moderate content of phytanic acid (0.24 wt%) and "yellow" silage aiming after a low content of phytanic acid (0.13 wt%). Thus, we have investigated how the intake of two different butter and cheese-types, affect plasma phytanic acid concentration in humans. In addition, we investigate if intake of milk fat from the green fed cows, affects the risk markers of the metabolic syndrome differently from milk fat from cows fed predominantly non-green feed.

## Subjects and Methods

### Study design

We conducted a 4 week, double-blind, randomized, parallel intervention study. In the intervention period subjects were supplied with diets that differed in FA composition, especially phytanic acid, by the Department of Human Nutrition. Participants were stratified according to baseline phytanic acid concentration in plasma and sex into two treatment groups receiving either a diet with a higher content of phytanic acid, phytanic acid group, than the control diet with a low content of phytanic acid, control group. We assessed all outcome variables at the start and end of the intervention period.

### Subjects

Male and female subjects, age 20 to 42, were recruited by advertising in local newspapers. The baseline characteristics of the 14 subjects who completed the study were not different between the two groups (Table 1). Exclusion criteria were: BMI > 30 kg/m^2^, current or previous chronic disease, regular use of medication, or drug and alcohol abuse. All participants were apparently healthy as indicated by a medical and lifestyle questionnaire. They all agreed to refrain from taking any dietary supplements, from donating blood 2 months before and during the study, and from taking any medication that might interfere with study measurements. All subjects were instructed to maintain the same level of physical activity throughout the study. The subjects completed a 3-day weighed-food record before the intervention and after two weeks of intervention, in order to assess potential differences in dietary intake between the groups before and during the intervention. All records were coded before being evaluated by a clinical dietician, who also calculated energy intake and dietary composition using a national database (Dankost; National Food Agenty, Søborg, Denmark). Mean habitual energy intakes were 10.3 MJ/d (range: 6.7-15 MJ/d); 30.9% (23.7-37.8%) of energy was from fat, 15.1% (11.8-18.1%) was from protein, and 52.2% (42.6-63.7%) was from carbohydrates. There were no significant different between the 2 groups in habitual dietary intake. The protocol and aims of the study were fully explained (orally and in writing) to the participants, who gave written informed consent. The Scientific-Ethical Committees for Copenhagen and Frederiksberg approved the research protocol (H-B-2009-052).

**Table 1:** Baseline characteristics for the 14 healthy subjects participating in the 4 week intervention^1^

	Treatment	
	
	Control group (n = 5)	Phytanic acid group (n = 9)
Women, *n*	2	6
Age, *y*	31.6 ± 10.3 (20-42)	28.3 ± 5.2 (21-36)
Height, *cm*	174.6 ± 12.8 (158-190)	171.5 ± 6.9 (162.5-181.5)
Weight, *kg*	68.7 ± 10.9 (56.1-80.25)	65.5 ± 9.5 (54.20-82.95)
BMI, *kg/m2*	22.4 ± 1.7 (20.36-24.5)	22.2 ± 2.1 (19.7-26.2)
Phytanic acid, *μM*	3.35 ± 1.49 (1.75-5.41)	2.93 ± 0.85 (1.89-4.12)
Glucose, *mmol/L*	5.53 ± 0.6 (5.11-6.58)	5.29 ± 0.33 (4.81-5.88)
Insulin, *pmol/L*	22.68 ± 11.88 (7.2-39.1)	29.86 ± 24.58 (7.2-86.6)
TAG, *mmol/L*	0.76 ± 0.35 (0.44-1.31)	0.88 ± 0.22 (0.63-1.32)
Total CH, *mmol/L*	4.22 ± 1.01 (3.14-5.38)	4.23 ± 0.83 (2.92-5.87)
LDL CH, *mmol/L*	2.39 ± 0.72 (1.67-3.24)	2.35 ± 0.54 (1.7-3.55)
HDL CH, *mmol/L*	1.41 ± 0.3 (1.04-1.83)	1.47 ± 0.3 (0.91-1.91)
CRP, *mg/L*	0.54 ± 0.96 (0,05-2.26)	1.25 ± 0.86 (0.05-2.62)

^1^Values are mean ± SD; range in parentheses. CPR, C-reactive protein; CH, Cholesterol; TAG, Triacylglycerol. There were no significant differences between baseline values in the 2 groups (ANCOVA).

### Production of test milk

Milk for the human study was produced by the experimental organic herd at Aarhus University by 56 Danish Holstein cows with an average daily milk production of 39 kg with 4.46% fat. The cows were divided into two groups and fed a concentrate consisting of 1:1 mixture of oat grain and rapeseed cake with 11% fat. This concentrate constituted 40% of the diet. The remaining 60% of the diet consisted of two types of silage, a "green" silage aiming after a high content of phytanic acid and a "yellow" silage aiming after a low content of phytanic acid. The green silage consisted of a mixture of white clover grass and alfalfa silage, while the "yellow" silage consisted of a mixture of corn silage, pea-barley whole crop silage with a small proportion of white clover grass silage. Feeding of the cows was initiated in November 2008 and milk was collected from both groups over one week in February. The milk was transported to Thise Dairy (Roslev, Denmark), where it was processed into butter and cheese. The higher intake of chlorophyll-containing "green" silage resulted in a phytanic acid content of 0.24 wt% in the butter and cheese used for the phytanic acid group compared to the control group were the "yellow" silage resulted in a phytanic acid content of 0.13 wt%.

### Diets and test fats

During the intervention period part of the diet of the subjects was replaced with test foods, butter and cheeses with differing FA compositions, provided by the Department of Human Nutrition (Table 2). The test food was butter and cheese with different concentration of phytanic acid. The fatty acid (FA) composition of the test fats in butter are presented in table 2. The FA composition in the two test fats differed only slightly and phytanic acid differed with 0.11 wt%. The test butter with the highest content of phytanic acid, also had the highest content of α-linolenic acid and a lower n-6:n-3 ratio of about 1.8. This is in agreement with the higher proportion of clover and grass in the green feeding regime. The butter and cheese were incorporated into buns and each day during the intervention the subjects were provided with 3 buns each day; two buns with butter and one with cheese. The buns contained 4.1 MJ of energy, with 10 E % from protein, 51 E % from carbohydrates, and 39 E % from fat (47.9 g). All buns contained 13 g of butter each; in addition the cheese bun also contained 32 g of 45+ cheese. This amount of butter and cheese yielded a fat intake from the test diet of 45 g a day (17.5 MJ), which corresponded to approximately 50% of the maximum recommended daily intake of fat for a subject with a daily energy requirement of 10 MJ. To avoid drop outs we decided to use test fat from both butter and cheese.

**Table 2:** Fatty acid composition of the two test butter

	Butter	
	
	Control (wt%)	Phytanic acid (wt%)
6:0	1.52	1.51
8:0	1.04	1.2
10:0	2.35	2.31
12:0	2.66	2.60
14:0	9.89	10.07
14:1	0.66	0.62
15:0	0.89	0.91
16:0	22.55	24.80
16:1n-7	1.15	1.16
Phytanic acid	0.13	0.24
17:0	0.47	0.50
17:1	0.20	0.20
18:0	15.39	16.00
18:1n-9 *trans*	2.59	2.25
18:1n-9 *cis*	25.67	25.17
18:1n-7	0.92	1.31
18:2 n-6 *trans*	0.49	0.47
18:2 n-6 *cis*	1.53	1.48
20:0	0.35	0.27
18:3 n-6	0.00	0.00
18:3 n-3	0.48	0.83
≥20:0	0.69	0.29

### Compliance

We used the concentration of pentadecanoic acid (15:0) in plasma as an indicator of milk fat intake, and thus of compliance, because 15:0 in serum is considered as a valid marker for intake of milk fat in humans [20-22]. In addition dietary records were used to reinforce the dietary advice and strengthen compliance, and the results of the last records were used in the calculation of dietary changes during the study.

### Blood sampling and analysis

After a 12-h overnight fast, venous blood was collected before the intervention period (day 1) and at the end of the intervention (day 28). Blood for FA analysis was collected into tubes containing EDTA, which were kept on ice, and the samples were centrifuged at 4°C and 2200 × g for 15 min.

All samples were stored at -80°C until the samples were analysed. All samples were analyzed at the Department of Human Nutrition in Copenhagen, except from the samples of phytanic acid and FA, which were analyzed at the Technical University of Denmark (DTU).

### Blood lipids

We assessed serum LDL and HDL cholesterol by enzymatic colorimetric procedure (ABX Pentra LDL Direct CP and ABX Pentra HDL Direct CP respectively) on ABX Pentra 400 Chemistry Analyzer (HORIBA ABX, Montpellier, France). Total cholesterol was assessed and analyzed by enzymatic photometric procedure (CHOD-PAP from ABX Pentra Cholesterol CP) on ABX Pentra 400 Chemistry Analyzer (HORIBA ABX, Montpellier, France). The concentration of TAG was assessed and analyzed by enzymatic colorimetric procedure (ABX Pentra Triglycerides CP) on ABX Pentra 400 Chemistry Analyzer (HORIBA ABX, Montpellier, France).

### Fatty acid analysis in lipids and test fats

Total lipids were extracted and analyzed from blood plasma samples as described by Tholstrup *et al *[23] but using TAG C19:0 as internal standard. The test fats/butters lipids were extracted according to the method of Folch [24], while preparation of FAME was performed as described earlier [23]. Response factor was calculated for methyl esters of phytanic acid and short-chained fatty acid, based on the response of palmitic acid methyl ester (C16:0). The butter FAME were separated on a 60-m Supelco SP-2380 column (Sigma-Aldrich AS, Brøndby, Denmark) in a HP 6890 gas Chromatograph (GC), in split mode using He as carrier gas. GC settings were: Injector temperature 260°C, split ratio 20: 1, carrier flow 1.2 ml/min, detector temperature 300°C, air flow in detector 300,0 ml/min, hydrogen flow 35 ml/min. FAME were separated using a temperature program starting at 50°C and rising to 160°C at 15°C/min; this temperature was kept for 0 min, hereafter the temperature was raised to 182°C at 1°C/min, and directly the temperature was raised to 200°C at 10°C/min, and the oven was kept at 200°C for 15 min before the temperature was raised to 225°C. The final temperature was kept for 12 min (total runtime 61.97 min). FAME was identified with authentic standards, and FA masses were determined based on the peak areas, compared with the internal standard. The commercial standard for phytanic acid was partially separated into two peaks, based on the stereoisomeric properties of phytanic acid. Both peaks were identified in the butter, as well as in the plasma. The separation was however not good enough to quantify the distribution between the isomers. The phytanic acid metabolite pristanic acid, was also separated in the method, this fatty acid did however not reach above the detection limit in any sample analyzed.

### C-reactive protein

Blood for analysis of C-reactive protein was collected into dry tubes; after samples were centrifuged at 2200 × g for 15 min at 20°C. Serum was stored at -80°C until the samples were analysed. The CRP concentrations were measured by using latex immunoturbidimetry (ABX Pentra CRP CP) on ABX Pentra 400 Chemistry Analyzer (HORIBA ABX, Montpellier, France).

### Glucose and Insulin

Blood for analyses of insulin and glucose concentrations was collected into dry tubes and fluoride citrate respectively; after coagulation, the samples were centrifuged at 2200 × g for 15 min at 20°C. The samples were stored at -80°C until the there were analysed. Insulin concentrations were measured in serum with a chemiluminescent immunometric assay on DPC Immulite 1000 (Siemens Medical Solutions Diagnostics, USA). The intra-assay CV% for insulin was 4.73%. Glucose concentration was measured in plasma and analyzed by enzymatic colorimetric procedure (ABX Pentra Glucose HK CP) on ABX Pentra 400 Clinical Chemistry Analyzer (HORIBA ABX, Montpellier, France).

### Statistical analysis

We used an analysis of covariance (ANCOVA) to compare effect of the two diets. The respective baseline values were used as covariates, and the analyses were thus adjusted for the baseline values of each response variable. When necessary values were log transformed to normalize the distribution of residual variance and to obtain variance homogeneity. Statistical tests were performed on the transformed data. Transformation was necessary to ascertain the concentrations of HDL cholesterol and CRP. SAS statistical software (version 9.2; SAS institute Inc, Cary, NC) was used for all statistical analyses. Data describing the characteristics of the participants are summarized as mean ± SD, and data on outcome variables are expressed as least squares (LS) means ± SEM, adjusted for baseline values. We tested baseline, age, BMI, phytanic acid and 15:0 for influence on the results, but all these parameters were not include in the statistical model, because no influence on results were found.

## Results

### Dietary intake and body weight

No significant changes in body weight and habitual dietary intake were observed during and after the intervention period. We excluded 4 food records (2 from each group) from wk 2 of the intervention from the statistical analysis. The distribution (% energy) of protein (P = 0.7180), carbohydrates (P = 0.7157), and total fat (P = 0.0774) did not differ between the groups.

### Compliance

The increase of serum15:0 was highest in the control group in which an average increase of 31% (p < 0.01 vs. baseline) was observed compared to an increase of 20% (p < 0.05 vs. baseline) in the phytanic acid group. Althoug, there was no difference in 15:0 between the two groups (p = 0,139), the higher increase could indicate a higher consumption of dairy products during the intervention in the control group.

### Blood samples

There tended to be a slight difference in plasma phytanic acid (P = 0.073) between the two groups (Table 3). The study was not designed to compare baseline and treatment, however, due to the explorative approach we considered important to report that the results showed a significant increase (P < 0.05) of plasma phytanic acid within both groups. Noteworthingly, the increase was highest in the control group where an average increase of 24% was observed compared to an increase of 15% in the phytanic acid group. There were no effects of the two treatments on serum total (P = 0.700), LDL (P = 0.274) and HDL (P = 0.475) cholesterol, the triacylglycerol (P = 0.202), CRP (P = 0.4043), serum insulin (P = 0.450) and serum glucose (P = 0.8126) (Table 3).

**Table 3:** Effects of the 4-wk dietary intervention^1^

	Treatment	
	
	Control group (n = 5)	Phytanic acid group (n = 9)
Phytanic acid, *μM*	4.16 ± 0.24	3.56 ± 0.18
Glucose, *mmol/L*	5.37 ± 0.10	5.34 ± 0.08
Insulin, *pmol/L*	33.05 ± 5.69	28.11 ± 4.22
Triacylglycerol, *mmol/L*	1.15 ± 0.15	0.9 ± 0.11
Total cholesterol, *mmol/L*	4.46 ± 0.2	4.56 ± 0.15
LDL cholesterol, *mmol/L*	2.59 ± 0.14	2.82 ± 0.10
HDL cholesterol, *mmol/L*	1.28 ± 1.10	1.41 ± 1.07
CRP, *mg/L*	0.26 ± 2.05	0.58 ± 1.77

^1 ^All values are LSmeans ± SEM. CRP, C-reactive protein. ANCOVA; baseline values were used as covariates.

## Discussion

In the current study we investigated to what extent milk fat from cows fed either "green" or "yellow" silage affects the concentration of phytanic acid in plasma in healthy young men and female. The intake of "green" silage resulted in a phytanic acid content of 0.24 wt% of total test fat compared to 0.13 wt% in the control group. The test fat was produced to mimic the variation we have seen in dairy fat on the Danish market, with low concentration of phytanic acid during the winter and highest concentrations late in the grazing season.

The main finding of this study was a significant increase of plasma phytanic acid within both groups, regardless of cows feeding regime and test diet phytanic acid content. The higher increase in plasma phytanic acid in the control group compare to the phytanic acid group is opposed to what we have expected. This could be due to different compliance and/or random differences in phytanic acid metabolism between the groups. Since phytanic acid is not produced endogenously in human [25], the presence in the human body is of exogenous origin and ingested from the diet almost exclusively as preformed phytanic acid [26]. The exogenous origin of phytanic acid in human makes it reasonable to assume, that the increased concentration of phytanic acid found after the intervention, was due to an increase intake in this period. When the concentrations of phytanic acid in serum from 250 healthy humans were analyzed, it ranged from 0.04 to 11.5 μM [27]. The large variation probably may illustrate that dietary habit are determining the concentration of phytanic acid. However, genetic factors in regard to the efficacy by which phytanic acid is metabolized may also play a role [11,18]. Because the test diet substituted only a part of the habitual diet the phytanic acid content in the test diets cannot be regarded as being the sole source of dietary phytanic acid. The low degree of control of exposure to additional dietary phytanic acid intake could potentially also have influenced the plasma phytanic acid concentration in the subjects. Indeed, it may have contributed to the surprisingly high increase of plasma phytanic acid within the control group compare to the phytanic acid group. This is supported by the higher average increase of 15:0 in the control group which seems to indicate a higher consumption of dairy products during the intervention compare to the phytanic acid group. In addition, the control group had a higher mean phytanic acid and 15:0 concentrations at baseline (data not shown), indicating a higher habitual dairy fat intake, which also could explain the higher concentration of phytanic acid after the intervention. A second source for phytanic acid is marine food, but the diet protocols of the subjects did not indicate any difference in habitual intake or during the intervention.

Although we in this study demonstrate that intake of dairy products with a low concentration of phytanic acid increase plasma phytanic acid concentrations, there was unfortunately no significant difference between the two groups. This may be related to the small number of subjects, higher baseline phytanic acid levels in the control group, higher consuming of dairy fat in the non-test component of the diet in the control group or a combination of the above. The modest difference in phytanic acid concentration in the test diets may not be considered to be very high but the differences are relevant since it is similar to the seasonal variation occurring in Danish dairy products. The current study does not give indication of any possible effects of plasma phytanic acid on the metabolic variables and potential risk of developing the metabolic syndrome.

Although the two test butters also differed in the n-3/n-6 ratio, due to a higher content of α-linolenic acid in the phytanic acid rich butter, we do not consider this differences relevant for the outcome of the intervention, since intake of linoleic and α-linolenic acid from the butters only contributed minimally to the test subjects total intake of these fatty acids. Thus, intake of the control butter contributed with only about 675 mg linoleic and 215 mg α-linolenic acid per day and the phytanic acid rich butter with the same amount of linoleic acid but with 380 mg α-linolenic acid. Since a typical daily Danish intake of polyunsaturated fatty acids for men and women are 14 and 10 g/day, respectively [28], the contribution from the test-butters have only marginally affected the total intake.

In the present study population, the plasma phytanic acid concentration varied between 1.75 and 6.13 μM. The EC_50 _for transactivation of all three RXR - isoforms by phytanic acid have been determined to be between 2.3 and 4 μM [9,10]. This indicate that plasma phytanic acid concentration in this study population occur in a concentration in which it has physiological relevance as a modulator of RXR-activity. Finally, the issue on the association between prostate cancer risk and phytanic acid intake needs to be taken into consideration. It has been hypothesized that phytanic acid could be associated with increased risk of prostate cancer [3]. However, no conclusive adverse effects of phytanic acid intake and concentrations within the normal range in healthy humans have been established and there is no data in the literature indicating that the modest changes in phytanic acid that are induced in present study, would have negative impact on human health [29]

The strengths of our study include its controlled and randomized design, follow-up of dietary compliance during the study, the use of validated dietary marker to monitor the dairy fat intake, the test fat was modified from milk originating from dairy cattle from the same herd, and the differences between the two types of test fat was obtained through natural reproducible cow feeding regimes. Furthermore the controlled cow feeding procedure is also considered to be an advantage of the study design. However, since the feeding regimes were designed to mimic what is possible to perform in Danish high-yielding herds, it was not possible to keep the cows on pure green feeding-regime. It would therefore be interesting to study the effect of dairy fat that are derived from cows fed pure green material (e. g. grazing cows), which would result in substantially higher concentration of phytanic acid, as well as other bioactive fatty acids. Among the limitations were the relatively high intakes of butter during the intervention. However, the increase in dietary fat during intervention did not differ between the two groups, and neither did the body weight after the intervention. It is also possible that difference in random habitual dietary habits and compliance such as and higher increase in serum 15:0, indicating higher dairy intake, in the control group after the intervention may have affected the results.

It would be relevant to use pure phytanic acid, but this has not yet been possible due to high costs required to produce purified synthetic phytanic acid products for human intervention studies. There is a general lack of evidence to support the results obtained in the study as research on phytanic acid effect in human is missing. Further research must elucidate if increase intake of dietary phytanic acid might play a part in improving the metabolic syndrome and increase the risk of prostate cancer in human.

## Conclusion

This study indicate that increased intake of dairy fat modify the plasma phytanic acid concentration, regardless of cows feeding regime and the minor difference in dietary phytanic acid content after 4 weeks. Further feeding-studies, in which phytanic acid is used in physiological relevant concentration, is essential before it is possible to conclude whether increased intake of phytanic acid have preventive effect on the metabolic syndrome in human.

## Abbreviations

RXR: retinoid-X receptor; PPAR-α: peroxisome proliferator-activated receptor-α; UCP 1: uncoupler protein 1; FA: fatty acid; 15:0: pentadecanoic acid; CRP: C-reactive protein

## Competing interests

The authors declare that they have no competing interests.

## Authors' contributions

LBW, MR and RAP carried out the practical work with the human study. LBW preformed the statistical analysis, and drafted the manuscript. LIH and TD analyzed the data and SKJ provided the milk for the project. TT designed the research.

All authors have read and approved the final manuscript.
